# The underestimated role of pioneering women in radiation oncology: lessons from the past for today’s practice

**DOI:** 10.1007/s00066-025-02386-z

**Published:** 2025-03-07

**Authors:** Michael Oertel, Vina Zielonka, Uwe Busch, Uwe Haverkamp, Maike Trommer, Angela Besserer, Franziska Eckert, Jilada Wilhelm, Rita Engenhart-Cabillic, Hans-Georg Hofer, Hans Theodor Eich, Oliver Micke

**Affiliations:** 1 Department of Radiation Oncology, University Hospital Münster, Albert-Schweitzer-Campus 1, building A1, 48149 Münster, Germany; 2 Institute for Ethics, History and Philosophy of Medicine, University of Münster, Münster, Germany; 3 German Roentgen Museum, City of Remscheid, Remscheid, Germany; 4 Department of Radiation Oncology, Cyberknife and Radiotherapy, Medical Faculty, University Hospital Cologne, Cologne, Germany; 5 Center for Integrated Oncology (CIO), Faculty of Medicine and University Hospital Cologne, University of Cologne, Cologne, Germany; 6 Austin Health, Department of Radiation Oncology, Olivia Newton-John Cancer Wellness & Research Centre, Melbourne, Australia; 7 Department of Radiation Oncology, Ernst von Bergmann Hospital Potsdam, Potsdam, Germany; 8 Department of Radiation Oncology, Medical University of Vienna/AKH, Vienna, Austria; 9 Department of Radiation Oncology, University Hospital Dusseldorf, Dusseldorf, Germany; 10 Department of Radiotherapy and Radiation Oncology, University Hospital Giessen-Marburg, Marburg, Germany; 11 Department of Radiotherapy and Radiation Oncology, Franziskus Hospital, Bielefeld, Germany

**Keywords:** History of radiation oncology, Women in medicine, Female pioneers in science, Radiation therapy, Gender disparities

## Abstract

**Purpose:**

The early history of radiation and radiation oncology is imprinted by innovative pioneers both in physics and clinical application. Despite the remarkable example of Marie Curie, the contributions of female physicians, physicists, and radiation therapists in the first years of radiation practice are often forgotten or neglected. This analysis aims at a comprehensive review of pivotal female pioneers in the field of radiation oncology and summarizes current and future challenges with regard to gender equality in the radiation oncology workforce.

**Methods:**

The History and Women in Radiation Oncology working groups of the German Society for Radiation Oncology (DEGRO) conducted a selective literature research on Marie Kundt, Marietta Blau, Elisabeth Fleischmann, and Anna Hamann, who were chosen as representative examples of female pioneers. Medical and sociological analyses were selected to illustrate the present situation and point out future challenges.

**Results:**

The review illustrates that women in radiation oncology in the late 19th/early 20th century were hindered in enrolling in educational institutions and in pursuing an equal (and recognized) professional career; they were also subject to discrimination. Thus, great dedication and personal sacrifices were needed to succeed. Despite this, significant contributions were made by women, and the four discussed colleagues contributed to or even enabled the formation of critical aspects of modern radiation oncology, such as X‑ray imaging, radiation physics, different treatment techniques, and the profession of radiation technicians. Lacking adequate radiation protection at the time, their inspirational spirit came at a significant cost, and three of the four presented pioneers (MB, EF, and AH) succumbed to irradiation-induced cancers. Today, modern analyses still show that female professionals tend to drop out during their career before professorship or head of department positions and are therefore underrepresented in these career stages.

**Conclusion:**

The history of women in radiation oncology is marked by discrimination and great personal and professional sacrifices. Despite these challenges, female pioneers contributed to the development of modern radiation oncology in a significant way. Today, gender disparities in the workforce persist and constitute challenges which need to be addressed to enable equal access to leading positions.

“Nothing in life is to be feared, it is only to be understood. Now is the time to understand more, so that we may fear less.” Marie Curie

## Introduction

It is quite easy to determine the beginning of the history of radiology and radiotherapy with the discovery of X‑rays by Wilhelm Conrad Roentgen (1845–1923) on November 8, 1895 [1], and the first radiation treatment by Leopold Freund (1868–1943) in 1896, which was published in 1897 [2].

But when did women appear in the history of radiology and radiotherapy? With the awarding of the Nobel Prize in Physics to Marie Curie (1867–1934) in 1903 [3] by the Royal Swedish Academy of Sciences?

The worldwide spread of the sensational news of the discovery of X‑rays was primarily fueled by the publication of the X‑ray image of Anna Bertha Roentgen’s hand.

**Fig. 1:**
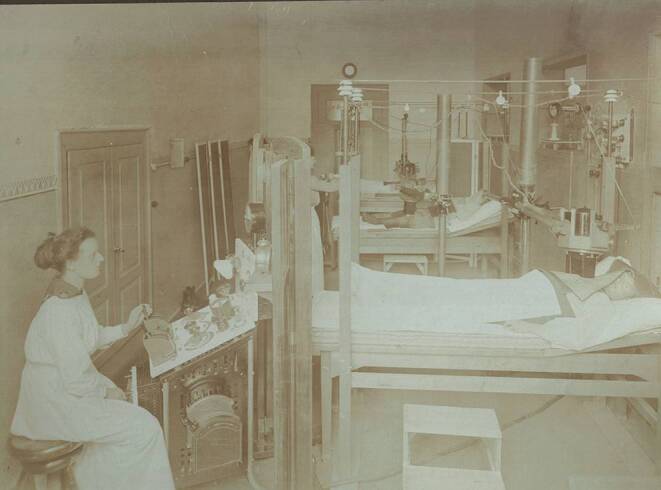
Radiation treatment room in the University Women’s Clinic Erlangen (around 1918; © Siemens Healthineers Med Archiv)

**Fig. 2:**
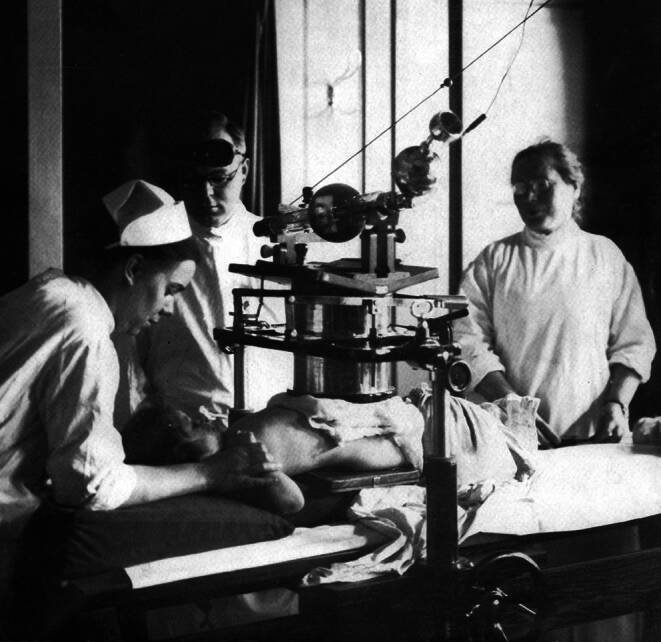
Pediatric X‑ray examination with compression aperture Berlin Moabit 1923 (© Sammlung Deutsches Röntgen-Museum)

**Fig. 3:**
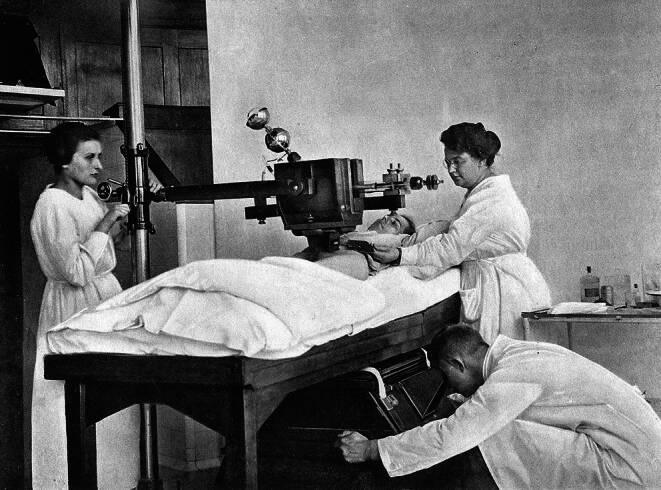
X‑ray therapy of the uterus in the University Women’s clinic Erlangen around 1920 (© Sammlung Deutsches Röntgen-Museum)

**Fig. 4:**
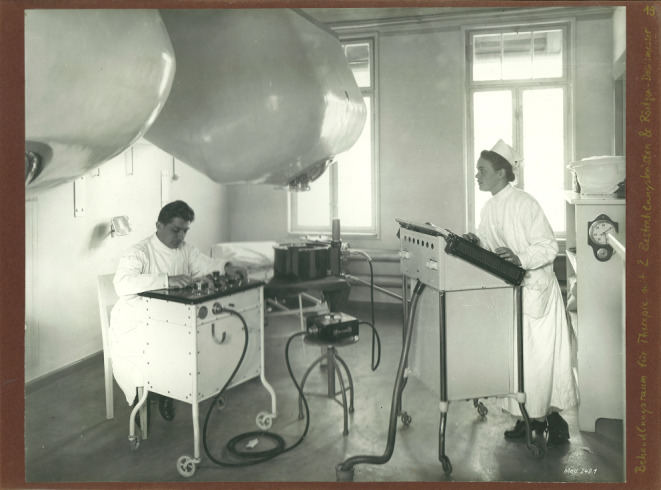
Siemens *Bestrahlungskasten* with dosimetry facility around 1919 (© Siemens Healthineers Med Archiv)

With the establishment of X‑ray facilities, essential tasks in the operation of the equipment, in the development of X‑ray plates, and in the implementation of radiotherapy were primarily carried out by women, who thus bore the main burden of work ([4]; Figs. 1, 2, 3 and 4).

Due to a lack of scientific knowledge, measures to protect doctors, nurses, and patients from ionizing radiation were usually completely neglected. Therefore, in the early days of radiology, female pioneers faced immense challenges and health risks due to their groundbreaking involvements in radiation sciences, including the discovery of radioactive elements and the development of techniques for medical imaging and cancer treatment [5].

Despite their important work, these women faced discrimination and marginalization in a male-dominated scientific community. They were denied access to an equal (university) education and often worked under difficult conditions with limited resources and support, putting their own health and wellbeing at risk in the pursuit of scientific knowledge and professional recognition.

In addition to professional challenges, women in radiology were impacted by broader social and political upheavals. During times of conflict and war, female radiologists, X‑ray nurses, radiographers, and radiological technicians bravely provided medical care and imaging services in challenging and dangerous environments, sometimes risking their own lives to save those of others [6, 7].

It is of pivotal importance to recognize that women have faced different types of sacrifices and struggles in their quest for knowledge, recognition, and equality. Their contributions to the field of radiology and radiotherapy are invaluable, and their stories can serve as inspiration for future generations of both women and men, to continue pushing the boundaries of scientific discovery and innovation [8].

To enlighten the broadness of women’s work in the early history of radiotherapy, we present the biographies of four different women whose lives reflect the whole width of radiation oncology: of an X‑ray nurse (radiation technician), a medical doctor, a physicist, and a technicians’ teacher. Even today, gender disparities persist in the radiation oncology workforce, which prompted our group to reflect on the current situation and present modern attempts to overcome inequality.

## Methods

The History and Women in Radiation Oncology working groups of the German Society for Radiation Oncology (DEGRO) conducted a selective literature research on Marie Kundt, Marietta Blau, Elisabeth Fleischmann, and Anna Hamann, who were chosen as representative examples of female pioneers. Medical and sociological analyses were selected to illustrate the present situation and point out future challenges.

## Gender disparities in the present radiation oncology workforce

In spite of inspiring examples of female pioneers, gender disparities persist across various domains of healthcare and medicine. The number of female medical students in Germany is increasing, and women constituted 64.3% of all medical students in 2022 [9]. Despite this fact, women tend to drop out disproportionately frequently with each career step, a phenomenon which is known as the “leaky pipeline” [10].

The concept of the leaky pipeline refers to the phenomenon where women and other underrepresented groups progressively leave their career paths at various stages. This metaphor highlights how women often get “lost” at various stages of the academic and professional career, resulting in significant gender disparities in higher-level positions [11].

Current data show the unbalanced ratio of women to men in leading positions in clinical disciplines at medical faculties of German universities [12]. In 2022, the analysis performed by the German Medical Women’s Association revealed a proportion of only 13% women in leading positions (Table 1). With 13% (2019) and 10% (2016) in prior analyses, no significant progress has been made.

**Table 1:** Overview on women in leading positions in medicine and radiation oncology. Respective percentage values according to the overall number of physicians

**Leading positions in medicine**	**Female percentage**		
Years	2016	2019	2022
Germany [12]	10%	13%	13%
**Women in radiation oncology**			
Years	2006	2020	–
United States [13]	23.4%	27.5%	
Germany (unpublished)	39%	–	
*Private lecturers*	0.7%		
*Professors*	0.3%		

These disparities also prevail in radiation oncology. In a cross-sectional study of different medical specialties in the United States, the percentage of female physicians in radiation oncology was estimated to be 23.4% (2006) to 27.5% (2020; Table 1; [13]). These numbers were below average and, with an increase of only 4.1% over time, represented the second lowest increase amongst all 16 specialties considered. Importantly, the authors identified the percentage of women within a specialty as a significant predictor for female medical students/residents pursuing this discipline. This trend translates into an underrepresentation of women in radiation oncology leadership positions [14]. However, the presence of at least one woman in a leadership position counteracts this effect and significantly increases the percentage of women among the faculty members (36.2% vs. 23.4% mean percentage of women in faculty in programs with one or more woman in a leadership position vs. those without; *p* < 0.001).

Within the DEGRO, a past analysis of all 1469 members from 2006 revealed 899 (61%) male members, with 65 (4%) private lecturers (*Privatdozent*) and 161 (11%) professors. In comparison, there were 570 (39%) female members, with only 11 (0.7%) private lecturers and 4 (0.3%) female professors (data not published). An update of this data is currently underway.

Conservative/nonsurgical disciplines like radiation oncology have been shown to be particularly vulnerable to negative impacts on education and training caused by the COVID-19 pandemic, as outlined by a recent survey amongst 450 participants, 28% of whom were radiation oncologists [15]. Participants from nonsurgical specialties described an increased workload and a discontinuation/reduction of in-house training significantly more often than participants from surgical disciplines. Furthermore, female participants were significantly more likely to report no access to in-house training (27%) and to indicate pressure due to excessive demands (31%). The long-term impact of the past pandemic is difficult to estimate and will manifest gradually in the years to come.

Addressing these issues requires not only policy changes but also a shift in the underlying discourses that shape our understanding of gender roles in professional settings.

Even today, radiation oncology is associated with common misconceptions like “radiation oncologists don’t see any patients” or “are unsociable and function in solitude,” which may prevent women from choosing this discipline [16]. The American Association for Women in Radiology (AAWR) Medical Student Outreach Subcommittee has started a social media campaign to address these misbeliefs and draw a more realistic picture of radiation oncology amongst medical students. However, a recent survey amongst undergraduate students in Germany revealed no gender-specific differences in the interest to pursue training in radiation oncology [17].

In Germany, the Women in Radiation Oncology Working Group (Frauen in der Radioonkologie, FiRO) constituted itself in April 2024 and will continuously work on multiple aspects of female representation and women’s empowerment in the radiation oncology workforce. Understanding the difficulties facing women in radiation oncology and the differences between men’s and women’s career paths is important to decrease obstacles and pave the way for a gender-equal future. The goal is to make radiation oncology more visible and appealing overall and to help to achieve more parity between women and men. The following four biographies illustrate the necessity of pursuing this goal.

## Female pioneers

### Marie Kundt—pioneer of scientific photography

**Fig. 5:**
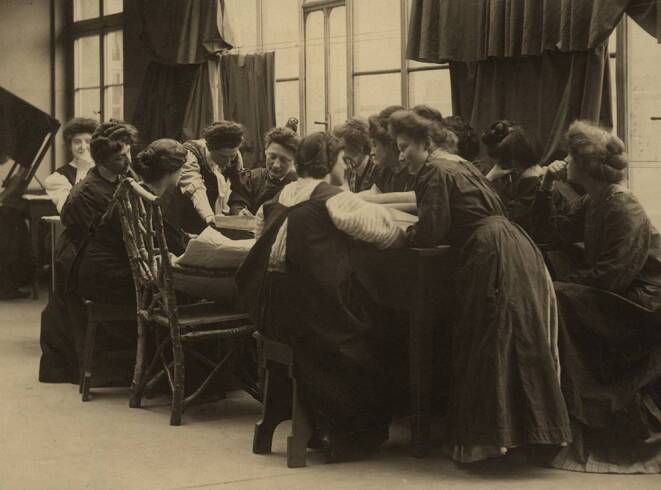
Lessons in the photography class of the Lette Verein; Marie Kundt is at the center with her face turned towards the observer. Undated (around 1911), by Anna Köppken. (Photography provided by courtesy of the Lette Verein, Berlin)

**Fig. 6:**
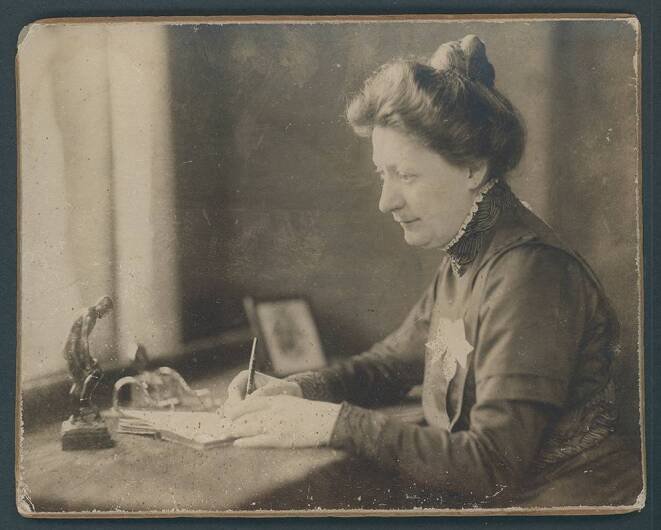
Portrait photography of Marie Kundt at her desk, around 1900 (unknown photographer). (Photo provided by courtesy of the Lette Verein, Berlin)

Marie Julia Berta Emma Kundt was born on February 4, 1870, as the second child of Helene Marie Elisabeth Kundt, born Willert, and Hans Carl Heinrich Kundt, who was an army captain in the East German state of Mecklenburg [18, 19]. The family’s military imprint (two of her brothers also served as officers) deeply influenced her life due to forced moves of the family but also owing to her proximity to a male-dominated domain [19]. This may have sparked her desire for an independent career. In addition, her uncle August Kundt (1839–1894) was a professor of experimental physics and the academic teacher of Wilhelm Conrad Roentgen. It was August Kundt who enabled her access to scientific circles [19, 20]. After having received two successful trainings as a drawing instructor and needlework teacher, she participated in the first photographic course of the Lette Verein in Berlin ([18, 19]; Figs. 5 and 6). The idea behind the Lette Verein was to provide independent professional opportunities for unmarried women at a time when women were not allowed to enroll at German universities [18, 19, 21, 22]. However, access to this institution was limited and costly [19]. At the time of the discovery of X‑rays in 1895, only 25 students were enrolled at the photographic institute [21]. After her exam in 1891, Marie Kundt worked as an assistant for the institution’s director Dankmar Schultz-Hencke (1857–1913). She later became his deputy in 1910 and succeeded as director in May 1913 [18, 19, 21]. She assisted in experiments with the innovative technology of X‑rays. In fact, the first X‑ray image acquired in Berlin showed the right hand of Marie Kundt [18, 19, 23]. The image was acquired with a 30-minute X‑ray exposition during a public demonstration with the physicist Eugen Goldstein (1850–1930); as none of the participants wanted to volunteer for the experiment, Marie Kundt had to courageously provide her hand and was even forced to put on a participant’s ring, as forgery with an already prepared image was suspected [23]. Marie Kundt conceptualized a 1-year curriculum covering major aspects of photography and thus contributed decisively to the development of the “scientific photographer” as a profession—an exclusively female occupation in hospitals, universities, and smelting works (men were granted access to this education 20 years later) [22]. This effort was the cornerstone of the professional formation of radiographers/radiological technologists, who soon became indispensable for medical care but also for the treatment of wounded soldiers during the war [18]. As head of the professional association Bund der Organisationen technischer Assistentinnen an wissenschaftlichen Instituten (BOTAWI) founded by her, she achieved a homogenous (and state-controlled) education and professionalization of technical assistants [18, 19, 21]. Her long-standing dedication to the formation of professional opportunities for women saw her awarded multiple honors by the Red Cross, the grand-duke of her home state, and the German empress [18, 21]. She passed away in Berlin on April 2, 1932, at the age of only 62 and was deeply mourned by her companions, her students, and also by political leaders [21]. Since 2013, an award of the Dachverband für Technologen und Analytiker in der Medizin (DVTA) for innovative achievements by medical technicians, such as improvements in quality control, radiation protection, diagnostics, or documentation, has been named after her [18, 19].

### Marietta Blau—discoverer of disintegration stars

When Roentgen discovered X‑rays in 1895, women were not allowed to pursue studies at the University of Vienna, a regulation which was abolished 2 years later [24–26]. Amongst the first female students in physics was Marietta Blau. She took on her studies in 1914 and completed her doctoral thesis in 1919, followed by internships, e.g., in the department of Guido Holzknecht (1872–1931) in Vienna. Afterwards, she moved to Berlin to work for an X-ray manufacturer, where she conducted electrotechnical and spectroscopical experiments. As she preferred an academic career to the industry, she applied for an assistant job at Frankfurt University. At the Institute for Medical Physics, she dedicated herself to the teaching of theoretical and practical basic knowledge of radiology for physicians.

In 1923, she returned to Vienna in fear for her mother and took on an unpaid job at the Institute for Radium Research of the Austrian Academy of Sciences. Such positions were unattractive to men but enabled upper-class women to pursue a career in science. This led to a high percentage of female scientists in the early years of research in radiation physics, some of whom (like Marie Curie) became very famous. This was, however, not the case for Marietta Blau, who was forced to fight for recognition by the scientific community. Her most important and best-known achievement is the discovery of “disintegration stars” together with her colleague Hertha Wambacher (1903–1950), whose dissertation she also supervised. Disintegration stars are tracks of reaction products induced by cosmic irradiation and visualized on a photographic emulsion [27, 28]. Blau had developed a special detection technique and conducted a series of experiments with photographic plates in the Alps which led to her discovery of disintegration stars. Her double-discrimination as both a woman and a Jew prevented her from receiving the public recognition she deserved [29]. Despite being nominated for the Nobel Prize in both physics and chemistry several times, she never received it. However, she was awarded the Schrödinger Prize of the Austrian Academy of Sciences in 1962 [30].

After the annexation of Austria by Nazi Germany in March 1938, Marietta Blau was forced to leave her home country due to her Jewish origin [31]. With the support of Albert Einstein (1879–1955), she was able to emigrate and got a lecturer’s position in Mexico City. Further job offers/positions brought her to Columbia University, New York, and the University of Miami, Coral Gables. Returning to Vienna for financial and health reasons in 1960, she continued her work at the Institute for Radium Research without any payment for another 4 years.

Marietta Blau became one of the many victims of radioactivity. The lack of adequate radiation protection in the Institute for Radium Research led to radiation damage to her hands, which caused her lifelong pain. In 1970, she succumbed to lung cancer in very poor economic conditions, like some of her coworkers at the institute before her. No obituary appeared in any scientific publication.

Despite her groundbreaking contributions, Blau’s achievements were largely overlooked during her lifetime. Her work was often credited to her male colleagues, and she did not receive the recognition or accolades she deserved. Largely forgotten after her death, Blau’s research achievements have only been rediscovered since the turn of the millennium [28].

### Elizabeth Fleischmann—a world-class radiographer

**Fig. 7:**
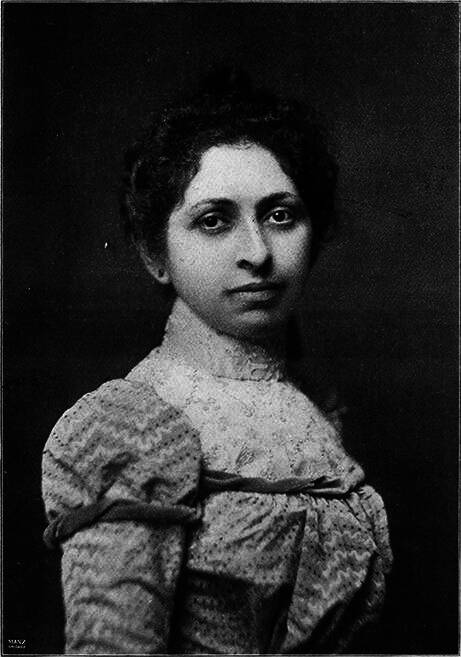
Portrait photography of Elizabeth Fleischmann by the *American X‑ray Journal*, around 1900 (public domain)

Elizabeth Fleischmann-Aschheim (1867–1905) was an American radiographer (Fig. 7). She is considered to be the first woman to die from X‑ray exposure [32, 33]. She was born on March 5, 1867, in California, into a family of Austrian Jewish immigrants just after the civil war [33, 34]. When the family moved to San Francisco in 1876, Elizabeth Fleischman attended the local girls’ high school. To support her family, she took accounting and office organization courses and worked as an accountant at an underwear manufacturer in San Francisco [33, 34].

Upon her mother’s death, Fleischman moved in with her sister Estelle who was married to the English physician and surgeon Michael J. H. Woolf. She worked as a bookkeeper in Woolf’s medical office. He encouraged her to study the new technology of X‑rays [34]. In 1896, she read about Roentgen’s discovery of X‑rays, which was reported in the morning edition of *Die Presse* in Vienna. Thereupon, she attended a public lecture and presentation of X‑ray machines by Albert Van der Naillen in San Francisco and enrolled at the Van der Naillen School of Engineering for electrotechnology [32–34].

After completion of her studies, her father helped her to purchase an X‑ray apparatus and a fluoroscope [32, 33, 35]. This was one of the very first privately owned pieces of equipment for the use of X‑rays in California. Fleischman examined patients on behalf of the local physicians [35]. She quickly became known as one of the most expert radiographers in the USA [32, 34, 36].

**Fig. 8:**
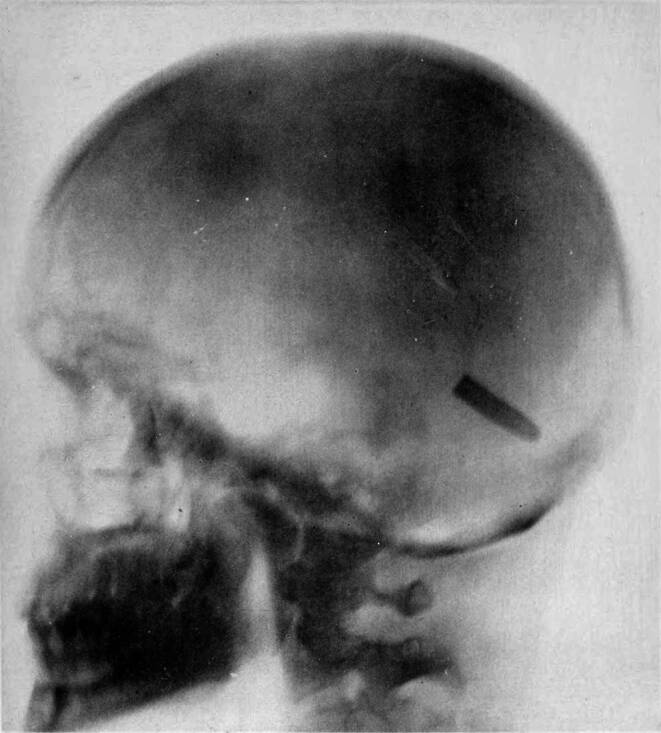
Radiograph by Elizabeth Fleischman (taken August 20, 1899) of the skull of Private John Gretzer Jr. with a bullet lodged in his brain in the region of the left occipital lobe [37]. (Public domain)

In December 1898, she began to work as a radiographer for the US Army, which was sending wounded soldiers from the Spanish–American War back to the USA via San Francisco [32–35]. The wounded men often carried unremoved bullets and shrapnel in their bodies. Fleischman located the bullets and splinters and thus helped Army surgeons to remove them properly [34, 35]. On August 20, 1899, she took one of her most famous radiographs, an image showing a Mauser 7 mm bullet lodged in the brain of John Gretzer Jr. in the region of the left occipital lobe (Fig. 8). When surgeon general George Miller Sternberg (1983–1915) of the American War Department saw Fleischman’s pictures, he was eager to meet her and give credit to her fine work [35–38].

**Fig. 9:**
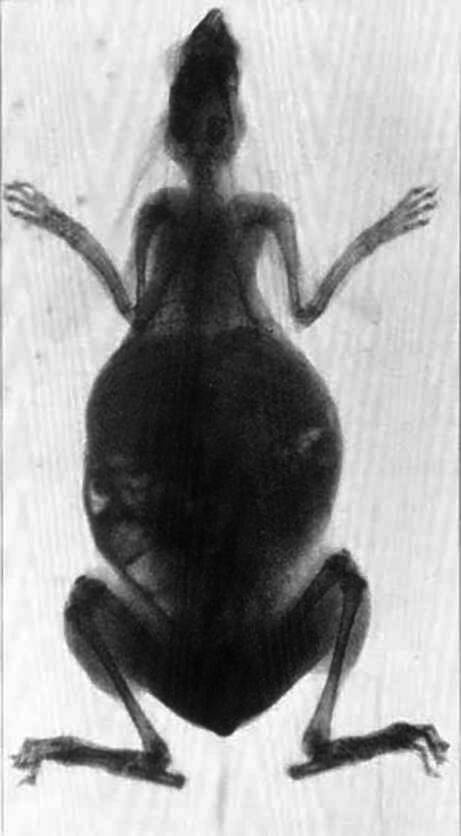
Belgian Hare (Camera Craft, June 1901 [39, 40]). (Plate by Elizabeth Kaufmann, public domain)

Fleischman also took X‑ray images of animals, like a rabbit (Fig. 9; [39]); food (in order to detect contaminations); and common objects, such as the interior of a shoe [34]. She elevated radiography to a new art form [40]. In 1900, she married Israel J. Aschheim, an emigrant from Prussia and assistant secretary to the California Board of Education, hyphenating her name to Fleischmann-Aschheim, which was very uncommon for a woman at that time [33].

The earliest radiology practitioners, like Elizabeth Fleischman, had no idea of the detrimental health effects that could result from continuous exposure to radiation [32–35]. Subjects were often exposed to massive doses of radiation, with exposure times between 20 minutes and several hours. In addition, X‑ray tubes were unshielded and uncollimated [35]. Radiographers often placed their own hands in front of the fluoroscope to check exposures. Moreover, Fleischman often exposed herself to X‑rays to show her patients that the procedure was absolutely painless [32–34, 41].

By 1903, the effects of nearly 7 years of unprotected X‑ray exposure and 12-hour workdays began to appear as X‑ray dermatitis on both of Fleischman’s hands. She attributed her symptoms to the chemicals used in developing photographic roentgen plates [35, 41]. In early 1904, the dermatitis progressed to the point where she sought medical assistance. She continued to work despite her injuries [42]. She introduced protective measures for the X‑ray machine operators like double-plated glass screens and metals such as lead, aluminum, iron, and copper. By late 1904, the dermatitis had progressed to cancer. Surgical tumor excision failed to stop the cancer progression. In January 1905, her entire right arm, including scapula and clavicle, had to be amputated. The cancer recurred 4 months later, and metastases were found all over her pleurae and lungs [32–35].

Elizabeth Fleischman finally died on August 3, 1905, at the age of 38. Throughout, she maintained her laboratory and X-ray practice [34, 35]. She had even started the experimental treatment of certain cancers with X‑rays. Taking note of her suffering, the obituary in the San Francisco Chronicle noted “Death came as a relief” [43].

Fleischman was the second person and the first woman to die as result of X‑ray exposure. The previous year, Clarence Dally (1865–1904), American glassblower and assistant and friend of Thomas Alva Edison in his work on X‑rays, had died under similar circumstances [32, 44].

Her tombstone on the Salem Cemetery in Colma outside of San Francisco bears the inscription: “I think I did some good in this world” [33, 34].

### Anna Hamann—leading scientist and head of department

Anna Hamann was born on July 14, 1894, as the only child of Mina Marie Marguerite and Carl Frederick Hamann, the latter being a physics professor at the University of Hamburg [45]. She graduated from Medical School in Munich in 1921 and completed her doctoral thesis on the “Development of Biological Foundations of Radium Therapy” 3 years later [46]. Early career steps of Anna Hamann included the Department of Radiology at Saint George’s Hospital in Hamburg under Hermann Holthusen (1886–1971), the Institut du Radium in Paris under Marie Curie (1867–1934), and the Radiumhemmet in Stockholm [45]. In 1929, she returned to Saint George’s Hospital in Hamburg as an associate assistant in radiology, where she intensified her research into radium. Her work focused, among other things, on the development of a photometric method for measuring radium doses and the “Hamburg method” [47] for treating uterine cancer [48, 49].

Hamann received board certification from the Specialty Board in Radiation Therapy in Germany (1928) as well as from the American Board of Radiology (1938) [45]. With her father being an active member of the German socialist party and therefore in opposition to the Nazi regime, Hamann was forced to leave Germany in 1938 [47]. She became an exchange professor at the University of Chicago. After a short return to Germany, Hamann was invited to reorganize the Department of Radiation Oncology at the University of Chicago [45]. From 1948, she chaired the Department of Radiation Therapy at Evanston Hospital, Illinois, until her retirement in 1962. She also worked as a consultant and associate professor at the Tumor Clinic at the Northwestern University Medical School and at the Swedish Covenant Hospital, Chicago [45].

Since her arrival in the United States, the FBI believed Anna Hamann to be a German spy, not only due to her origin, but also due to a suspicious feature: the loss of fingerprints after her radium experiments [50]. However, she continued treatment and experiments with radium, ignoring the extreme short sightedness which enabled her to identify persons only within millimeters of her eyes [50]. In 1969, she succumbed to subungual melanoma, a radiation-induced malignancy and consequence of her radiation experiments [50].

Anna Hamann was a member of the Deutsche Roentgen Gesellschaft, the American Medical Association, the Radiological Society of North America, the American Radium Society, the American Society of Therapeutic Radiologists, and the the American International Women’s Association, as well as being an honorary member of the Chicago Roentgen Society [45]. She was an internationally renowned researcher, an esteemed teacher, and a dedicated clinician. By this, she represents the archetype of an innovative and passionate physician who did not spare herself and her health in her pursuit of improving cancer care. Today, a scholarship by the DEGRO is named after her.

## Lessons for the future

The four women described in this article are exemplary for all those women in radiology and radiation therapy who have made significant sacrifices or faced substantial challenges throughout their careers. They dedicated themselves to the fields of radiology and radiotherapy, despite adversity and difficult circumstances. Their stories can teach us several important lessons for the future:

By their decision to serve science, the women consciously chose to go against society’s preconceptions of their role as a wife and a mother. Throughout their work, they faced severe challenges, including exposure to radiation risks before the dangers were fully understood [51]. They worked under conditions that put their health and lives at risk, yet they persisted in doing their work, thus contributing significantly to medical advancements. Their stories highlight the importance of dedication to one’s profession and the willingness to sacrifice oneself for the greater good. Of course, this character trait is not uniquely female and other pioneers in radiation oncology also pursued their scientific and clinical work regardless of any personal sacrifices, e.g., Guido Holzknecht [52].

However, these women were pioneers in a field that was not always welcoming of them. Their willingness to push boundaries, often as the first or only women in their department or specialty, shows a power of resilience and pioneering spirit. This teaches us the value of courage as well as the impact of trailblazing individuals for opening doors for future generations [53].

Their stories also underscore the importance of recognition and institutional support. Many of the women worked in environments where their contributions were not fully recognized or appreciated until much later. This highlights the need for institutions to actively recognize and support the contributions of all their members, particularly those at risk of being marginalized or overlooked [54].

The women’s sacrifices contributed to an increased awareness of the dangers of radiation exposure and the importance of radiation safety. In doing so, they were early pioneers not just in terms of advancing technology and clinical procedures but also in terms of enhancing safety measures and the wellbeing of later generations [55].

Their stories may serve as powerful inspiration for future generations in medicine. Moreover, we can learn about the importance of dedication, the power of pioneering spirit, the need for recognition and institutional support, the advancement of safety and awareness, and the inspirational impact on future generations. These lessons are crucial to the history of radiology and radiation oncology and to the promotion of gender equality and safety in the field.

## Conclusion

The four biographies presented in this article illustrate the challenges women faced in the early years of radiology and radiation oncology when establishing themselves as clinicians, scientists, technicians, or teachers in a male-dominated (professional) world. They consciously chose to go against society’s preconceptions of their role as a wife and mother in order to serve science—sometimes at the cost of premature death due to radiation damage. Their stories reveal women’s contributions to the development of radiation oncology and call on us, even today, to increase their visibility. Despite major improvements in gender parity and female empowerment, true professional equality demands continuous efforts in the present and future.
